# Antimicrobial Susceptibility Testing for *Corynebacterium* Species Isolated from Clinical Samples in Romania

**DOI:** 10.3390/antibiotics9010031

**Published:** 2020-01-16

**Authors:** Cristiana Cerasella Dragomirescu, Brandusa Elena Lixandru, Ileana Luminita Coldea, Olguta Nicoleta Corneli, Marina Pana, Andi Marian Palade, Violeta Corina Cristea, Ioana Suciu, George Suciu, Loredana Sabina Cornelia Manolescu, Loredana Gabriela Popa, Mircea Ioan Popa

**Affiliations:** 1 “Cantacuzino” National Medico Military Institute for Research and Development, 050096 Bucharest, Romania; ceraseladragomirescu@yahoo.com (C.C.D.); brandusa_lixandru@yahoo.com (B.E.L.); luminitacoldea@yahoo.com (I.L.C.); olgutza_dracea@yahoo.co.uk (O.N.C.); marina.pana@yahoo.com (M.P.); print_andi@yahoo.com (A.M.P.); mircea.ioan.popa@gmail.com (M.I.P.); 2 “Carol Davila” University of Medicine and Pharmacy, 050474 Bucharest, Romania; dr.gabriela.popa@gmail.com; 3 Central Reference Laboratory Synevo, 021408 Bucharest, Romania; violeta.cristea@synevo.ro; 4 BEIA Consult International, Peroni 16, 041386 Bucharest, Romania; ioana.suciu@beia.ro (I.S.); george@beia.ro (G.S.); 5 Colentina Clinical Hospital (CDPC), 020125 Bucharest, Romania

**Keywords:** *Corynebacterium* spp., antimicrobial susceptibility testing, multidrug resistance phenotypes

## Abstract

Antimicrobial resistance is one of the most important public health issues. Besides classical multidrug resistance species associated with medical care involved in superficial or invasive infections, there are strains less commonly associated with hospital or outpatient setting’s infections. Non-diphtheria *Corynebacterium* spp. could produce infections in patients with or without immune-compromised status. The aim of our study was to determine the susceptibility to antimicrobial agents to *Corynebacterium* spp. from clinical samples collected from Romanian hospitalized individuals and outpatients. Twenty Corynebacterium strains were isolated and identified as *Corynebacterium striatum* (*n* = 7), *Corynebacterium amycolatum* (*n* = 7), *C. urealyticum* (*n* = 3), *Corynebacterium afermentans* (*n* = 2), and *Corynebacterium pseudodiphtheriticum* (*n* = 1). All isolates have been tested for antibiotic susceptibility by standardized disc diffusion method and minimal inhibitory concentration (MIC) tests. Seventeen isolates demonstrated multidrug resistance phenotypes. The molecular support responsible for high resistance to quinolones for ten of these strains was determined by the detection of point mutation in the gene sequence *gyrA*.

## 1. Introduction

The Corynebacterium genus currently numbers around 129 species and subspecies isolated from human, mammals, and environmental sources [1]. Many species are members of the normal skin and the upper respiratory tract microbiota and can produce infections, especially in immune-compromised or debilitated human patients [2]. In the recent years, numerous studies have shown the involvement of several Corynebacterium species, other than *C. diphtheriae*, the classic pathogen of the genus, in superficial and invasive infections [3,4,5,6,7,8,9,10,11,12,13,14,15,16,17,18,19,20,21,22,23,24]. Due to the fact that the normal habitat for these species is the human skin and mucous membranes, they are therefore sometimes isolated as contaminants in clinical samples [2]. In recent times, the nontoxigenic strains have developed resistance to antibiotics, which makes it difficult for the treatment of infections [25].

To identify the species that belong to Corynebacterium genus, several types of methods are used: phenotypic, molecular, and mass spectrometry-based methods. Due to this variety of identifications possibilities, new species from the Corynebacterium genus have been isolated from clinical samples [1], such as the following that are presented below, the most frequent isolated species. Unfortunately studies about prevalence of the infections caused by these microorganisms, either in hospitalized patients or out-patients, are scarce. However, there are more and more articles that report hard to treat infections due to nontoxigenic strains of *Corynebacterium* spp. with resistance to antibiotics [6,19,22].

*C. amycolatum* was isolated from severe human infections such as bacteremia and endocarditis [3,4,5]. It was also encountered in acute/chronic complicated skin and soft tissue infection [26]. *C. amycolatum* isolates often display multidrug-resistance phenotypes to β-lactams, macrolides, clindamycin, aminoglycosides, quinolones, and rifampicin [6,27].

*C. striatum*, a species found in cattle, is part of human normal nose microbiota and is also a transient colonizer of the human skin. Since 1993, there have been reported cases of infection with *C. striatum* in immune-compromised individuals leading to the conclusion that this species can be considered an emerging pathogen for this group of patients [7,8,9].

*C. urealyticum* has been associated with acute or chronic infections of the urinary tract, urolithiasis, kidney stones and ureteral stenosis [10,11]. Although considered a microorganism involved in the pathology of the urinary tract, *C. urealyticum* has been isolated from blood culture, endocarditis, pericarditis, osteomyelitis, wounds, and soft tissue infections [12,13,14,15].

*C. urealyticum* strains are usually resistant to several β-lactams, aminoglycosides, and macrolides only a few isolates remaining sensitive to β-lactam antibiotics [16]. Susceptibility to fluoroquinolones is variable, the most active agent being ofloxacin. Doxycycline, rifampicin, and antibiotics belonging to the glycopeptides class (vancomycin and teicoplanin) are the most active drugs against this microorganism. In hospital settings, quinolones and rifampicin resistant clones are often selected after antibiotics therapy [16].

*C. afermentans* was isolated in a case of endocarditis, and it was found susceptible to ampicillin, cefazolin, ceftriaxone, gentamycin, erythromycin, ciprofloxacin, and imipenem, and resistant to clindamycin and trimethoprim-sulfamethoxazole [17].

A case of pleuropulmonary necrotizing infection was described in a patient infected with HIV in 2004 and the ethiologic agent was a subspecies of *C. afermentans* named *C*. *afermentans* subsp. *lipophilum*. Treatment with a combination of antibiotics over a long period of time leads to the remission of the infection without surgery [17].

*Corynebacterium pseudodiphtheriticum* is normally found in the oropharyngeal flora of the human respiratory tract. In immune-suppressed patients, infections associated with this species include exudative pharyngitis, bronchitis, bronchiolitis, necrotizing tracheitis, tracheobronchitis, pneumonia, and lung abscess [18,19]. It was also involved in the etiology of native valve endocarditis in patients with preexisting valvular lesions [20]. *C. pseudodiphtheriticum* is usually susceptible to β-lactams, vancomycin, and aminoglycosides being resistant to erythromycin, clindamycin, tetracycline and quinolones.

According to molecular studies, such as Alibi et al. study from 2017, in several *Corynebacterium* spp., double mutations leading to an amino acid change in positions 87 and 91 in the quinolone resistance-determining region of the *gyrA* gene point out that resistance to fluoroquinolones is frequently encountered in nontoxigenic strains of *Corynebacterium* spp. [28].

In Romania, the epidemiological data regarding the involvement of *Corynebacterium* spp. strains in infectious pathology are scarce. To the best of our knowledge, the only existing studies concern only a few isolates: three isolates of *Corynebacterium urealyticum* recovered from urinary tract disease in children, in 1997 [29], one strain of *Corynebacterium striatum* isolated from a lung infection [30], and a few *Corynebacterium striatum/amycolatum* strains isolated in ventilator-associated pneumonia at the Cardiovascular Surgery Clinic of Iaşi RI [31]. There are no data about antibiotic susceptibility patterns of these isolates.

The aim of our study was to establish the antibiotic susceptibility patterns in *Corynebacterium* spp. strains isolated from Romanian patients.

## 2. Results

The analyzed isolates were identified as *Corynebacterium striatum* (*n* = 7), *C. amycolatum* (*n* = 7), *C. urealyticum* (*n* = 3), *C. afermentans* (*n* = 2), and one isolate was classified as *C. pseudodiphtheriticum.* There were twenty samples tested. Fourteen came from admitted patients and the rest were from outpatients [21]. The origin of samples and the data resulted from antimicrobial susceptibility testing by MIC determination and disk-diffusion method is presented in Table 1.

Seventeen out of the twenty strains studied were resistant to penicillin G, cefuroxime, ceftriaxone, ciprofloxacin and chloramphenicol.

Sixteen strains out of the twenty were resistant to erythromycin. Fifteen strains out of the twenty were resistant to clindamycin and rifampin, whereas fourteen strains out of the twenty showed resistance to gentamicin. All twenty tested strains were susceptible to linezolid, vancomycin, and teicoplanin.

Two strains out of three of *Corynebacterium urealyticum*, six strains out of seven of *Corynebacterium striatum*, and one strain out of seven of *C. amycolatum* were resistant to nine out of the eleven antimicrobials tested and were sensitive only to the remaining two antimicrobials tested namely glycopeptides and linezolid. Six strains out of seven of *C. amycolatum* and two strains of *C. afermentans* (all *C. afermentans* strains) were resistant to eight out of eleven antimicrobials tested.

Six out of seven *Corynebacterium amycolatum* isolates were resistant to eight out of eleven antibiotics tested and one strain out of seven was resistance to nine out of eleven antibiotics tested. Six out of seven *Corynebacterium striatum* isolates and two out of three *Corynebacterium urealyticum* isolates were resistant to nine antibiotics tested.

**Figure 1:**
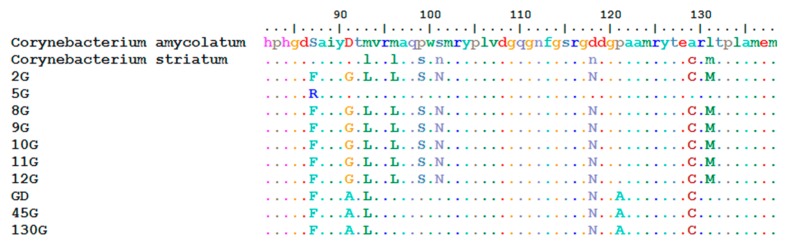
Demonstration of point mutations resulting in amino acid substitution at positions 87 or 91. Isolates codes 5G, GD, 45G, and 130G are from *Corynebacterium striatum* and the rest from *Corynebacterium amycolatum* spp.

For ten strains of *C. amycolatum* and *C. striatum*, we revealed the presence of two point mutations in the *gyrA* gene sequence, mutations responsible for high resistance to fluoroquinolones. The presence of these point mutations determines the change of amino acids in 87 and 91 positions of *C. amycolatum* and *C. striatum* strains, and it is responsible for high resistance to fluoroquinolones, see Figure 1.

## 3. Discussion

In Romania, many infections caused by *Corynebacterim spp.* often remain undiagnosed because these species (especially *C. urealyticum*) grow more slowly on usual culture media, or may be incorrectly classified. Also, the possible involvement of these microorganisms in nosocomial infections should not be underestimated, giving their multi-resistant pattern to frequently used antibiotics. The studies show that multi-resistant strains remain susceptible to glycopeptide antibiotics so far.

Funke et al. affirm this as follows. “Identification of coryneform bacteria to the species level often causes problems but should be performed whenever they grow in pure culture from clinical specimens and/or when they represent the predominant organisms in normally sterile samples” [2].

Before 2014, the MIC had to be determined, and although this is an accurate method and still recommended by CLSI standard, it is laborious, expensive, and inaccessible for routine antimicrobial susceptibility testing in many bacteriology laboratories.

Our study confirms specialized literature data [6,14,15,22,27]. The antibiotics that we tested belong to nine different classes and they are the most commonly used treatment options for *Corynebacterium* spp. infections (penicillins, cephalosporins, macrolides, aminoglycosides, phenicols, quinolones, ansamycins, tetracyclines, and glycopeptides). Our data on multidrug-resistant (MDR) phenotypes also confirm studies published in specialized foreign literature on *Corynebacterium* spp. MDR phenotypes [22,32].

*Corynebacterium* spp. isolates displaying multidrug resistance phenotypes originated either from hospitalized patients or from outpatients who declared no recent hospitalization. Their unusual multidrug resistance profiles could be explained by the frequent antibiotic ambulatory therapy.

The isolates from hospitalized patients could suggest a possible unrelated nosocomial transmission. Using as example the isolates collected in two different hospitals, isolate number 8 (Table 1) was recovered from surgical wound secretion (patient admitted to a county hospital) and the isolate 9 was recovered from a drainage catheter. In this case, a possible nosocomial transmission is sustained in our opinion by antimicrobial multidrug-resistant phenotypes. Using the same criteria, *C. pseudodiphteriticum* isolated from conjunctival secretion is probably a contaminant microorganism and not an etiological infection agent.

Vancomycin or vancomycin associated with rifampicin is frequently recommended as empiric therapy for invasive Gram-positive infection until susceptibility testing is performed [6]. Antimicrobial resistance for *Corynebacterium* spp. strains is often unpredictable and the determination of their susceptibilities is necessary for the best therapeutic results [6].

## 4. Materials and Methods

### 4.1. Bacterial Strains

Twenty *Corynebacterium* spp. identified as *Corynebacterium striatum* (n = 7), *C. amycolatum* (n = 7), *C. urealyticum* (n = 3), *C. afermentans* (n = 2), and one isolate *C. pseudodiphtheriticum*, isolated from blood culture (n = 6), peritoneal fluid (n = 2), urine (n = 3), wounds (n = 5), drainage catheter (n = 1), perirectal abscess (n = 1), osteomyelitis (n = 1), and conjunctival secretions (n = 1) were analyzed in Vaccine Preventable Diseases Laboratory, “Cantacuzino” National Institute for Research, Bucharest, Romania to determinate their susceptibility patterns to thirteen antibiotics. The bacterial growth of Corynebacterium strains was obtained on Columbia Blood Agar supplemented with 7% sheep blood, incubated aerobically for 24–48 h at 37 °C. The microbial identification was performed using API Coryne kit and confirmed by ARN 16S sequence analysis.

### 4.2. Antimicrobial Susceptibility Testing

Agar dilution methods were performed according to CLSI (Clinical and Laboratory Standards Institute) standards guidelines [33] for 10 antibiotics. The antibiotics tested were penicillin, erythromycin, cefuroxime, ceftriaxone, ciprofloxacin, chloramphenicol, gentamicin, rifampicin, tetracycline, and vancomycin. Antibiotic powders were provided from Sigma-Aldrich local distributor and were diluted in physiological saline solution or alcohol, according to the manufacturer’s instructions and CLSI recommendations [34] and were included in Mueller–Hinton medium with the addition of 10% sheep blood. For each antibiotic, six dilutions were performed including break points recommended by the CLSI standard and corresponding to the genus Corynebacterium (Table 2).

Ten microliters from 1/100 dilution of 0.5 McFarland bacterial suspensions (approximately 10^4^ UFC/spot) were applied on Mueller–Hinton Blood Agar using Steer applicator. All strains were tested for antimicrobial susceptibility to teicoplanin by E-test method according to the manufacturer’s instructions.

*Staphylococcus aureus* ATCC 29213 and *Streptococcus pneumoniae* ATCC 49619 were used for quality control.

In 2014, EUCAST introduced guidelines on antimicrobial susceptibility testing for *Corynebacterium* spp. strains, non-*C. diphtheriae*, using disk diffusion method [35].

McFarland bacterial suspensions (0.5) were spread on Mueller–Hinton Agar supplemented with 5% defibrinated horse blood. The antibiotics tested were: penicillin, clindamycin, ciprofloxacin, gentamicin, rifampicin, tetracycline, linezolid and vancomycin.

### 4.3. PCR Amplification of gyrA Gene

Each PCR reaction was performed using primers for *gyrA* gene described by Sierra et al. [32].

Reactions were carried out in 25 µL volumes with final concentrations/amounts of reagents of 0.1 µM each primer, 0.2 mM dNTP, 0.02 U/μL Taq, and 2 µL crude DNA extract. The polymerase used was Taq polymerase.

PCR conditions consisted of an initial activation at 94 °C for 2 min, followed by 30 amplification cycles at 94 °C for 30 s, 55 °C for 60 s, and 72 °C for 90 s, and a final extension at 72 °C for 7 min. PCR products were separated by agarose gel electrophoresis in a 2% (w/v) gel, using conventional gel electrophoresis.

For sequencing reactions, the *gyrA* PCR products (337 bplength) were purified using WIZARD SV Gel and PCR Clean Up System kit as recommended by the manufacturer.

The obtained amplicons were sequenced on both strands using the same primers, BigDye Terminator v 3.1 cycle sequencing kit on a 3100 Avant Genetic Analyser manufactured by ThermoFisher Scientific, Schwerte, Germany. The sequences were edited and aligned using the BioEdit version 7.0.5.3. software from Softpedia. Furthermore, the software can be deployed on cloud computing infrastructure and enable faster data processing [36].

Our analyzed isolates were collected during several years due to the rarity of the infections with nontoxigenic strains of *Corynebacterium* spp.

Our research was carried out with the approval and in accordance with the guidelines of the local Ethics Committee of University of Medicine and Pharmacy Carol Davila from Bucharest. All the procedures in the study respect the ethical standards in the Helsinki Declaration, and the protocol was approved by the Ethics Committee with the code 232/2019.

## 5. Conclusions

Our study demonstrates the presence of multidrug resistance *Corynebacterium* spp. in clinical isolates collected from hospitalized patients and outpatients in Romania. Seventeen isolates out of twenty tested demonstrated multidrug resistance phenotypes. The molecular support responsible for high resistance to quinolones for ten of these strains was determined by the detection of point mutation in the gene sequence *gyrA*.

The analyzed strains exhibit a wide and various resistances to many antimicrobial agents that were tested, therefore the evaluation of *Corynebacterium* spp. susceptibility.

These results are useful for microbiologists and clinicians, providing information on the involvement of these species in superficial or invasive infections and in choosing the best therapeutic decision in infections caused by these strains. It also raises attention over the existence of nontoxigenic strains of *Corynebacterium* spp. with resistance to antibiotics that are involved in potential life threatening pathologies.

Our study outline the need to introduce clinical antimicrobial therapies guidelines for *Corynebacterium* spp. involved in different infections in hospitalized or ambulatory patients in Romania. We consider that a multicenter study in several European countries regarding all types of *Corynebacterium* spp. is needed, due to the re-emergence of *Corynebacterium diphtheriae*, the toxigenic strain, in regions close to our country.

## Figures and Tables

**Table 1:** The results of antimicrobial susceptibility testing by MIC determination and disk diffusion method (Caption: S = Susceptible; R = Resistant). Isolates codes appear at strains with detected point mutations, see Figure 1.

No.	Sample	Species	P	CXM	CRO	E	CN	C	CIP	RA	TE	VA	TEC	DA	LNZ
1/2G	Blood culture I	*C. amycolatum*	R	R	R	R	R	S	R	R	R	S	S	R	S
2	Blood culture II	*C. afermentans*	R	R	R	R	S	R	R	R	R	S	S	R	S
3	Blood culture III	*C. afermentans*	R	R	R	R	S	R	R	R	R	S	S	R	S
4/5G	Blood culture IV	*C. striatum*	R	R	R	R	R	R	R	R	R	S	S	R	S
5/GD	Blood culture V	*C. striatum*	R	R	R	R	R	R	R	R	R	S	S	R	S
6/8G	Blood culture VI	*C. amycolatum*	R	R	R	R	S	R	R	R	R	S	S	R	S
7/9G	Peritoneal fluid I	*C. amycolatum*	R	R	R	R	R	R	R	S	R	S	S	R	S
8/10G	Peritoneal fluid II	*C. amycolatum*	R	R	R	S	R	R	R	R	R	S	S	S	S
9/45G	Catheter	*C. striatum*	R	R	R	R	R	R	R	R	R	S	S	R	S
10/11G	Osteomyelitis	*C. amycolatum*	R	R	R	R	S	R	R	R	R	S	S	R	S
11/12G	Perirectal abcess	*C. amycolatum*	R	R	R	R	R	R	R	R	R	S	S	S	S
12	Urine I	*C. urealyticum*	S	S	S	S	R	S	S	S	S	S	S	R	S
13	Urine II	*C. amycolatum*	R	R	R	R	R	R	R	S	R	S	S	S	S
14	Urine III	*C. urealyticum*	R	R	R	R	R	R	R	R	R	S	S	R	S
15/130G	Wound I	*C. striatum*	S	S	S	S	S	R	S	S	S	S	S	S	S
16	Wound II	*C. striatum*	R	R	R	R	R	R	R	R	R	S	S	R	S
17	Wound III	*C. urealyticum*	R	R	R	R	R	R	R	R	R	S	S	R	S
18	Wound IV	*C. striatum*	R	R	R	R	R	R	R	R	R	S	S	R	S
19	Wound V	*C. striatum*	R	R	R	R	R	R	R	R	R	S	S	R	S
20	Conjunctival secretion	*C. pseudodiphtheriticum*	S	S	S	S	S	S	S	S	S	S	S	S	S

Penicillin = P; Cefuroxime = CXM; Ceftriaxone = CRO; Erythromycin = E; Gentamicin = CN; Chloramphenicol = C; Ciprofloxacin = CIP; Rifampin = RA; Tetracycline = TE; Vancomycin = VA; Teicoplanin = TEC; Clindamycin = DA; Linezolid = LNZ.

**Table 2:** Break-point, minimal and maximal concentrations used for antimicrobial testing by MIC determination. (Caption: S = Susceptible; R = Resistant; I = Intermediate.)

Antibiotic	MIC (µg/mL) Criteria for the Interpretation of Antimicrobial Activity			Minimal Concentration (µg/mL)	Maximal Concentration (µg/mL)
	S µg/mL	I µg/mL	R µg/mL	µg/mL	µg/mL
Penicillin G	≤1	2	≥4	0.25	8
Cefuroxime	≤1	2	≥4	0.25	8
Ceftriaxone	≤1	2	≥4	0.25	8
Erythromycin	≤0.5	1	≥2	0.12	4
Gentamicin	≤4	8	≥16	1	32
Ciprofloxacin	≤1	2	≥4	0.25	8
Tetracycline	≤4	8	≥16	1	32
Rifampin	≤1	2	≥4	0.25	8
Chloramphenicol	≤8	-	≥8	1	32
Vancomycin	≤4	-	-	0.25	8
Teicoplanin	≤8	16	≥32	0.16	258
